# Supplementary material for: Inducing Kondo screening of vacancy magnetic moments in graphene with gating and local curvature
Source: Nat Commun. 2018 Jun 14;9:2349. doi: 10.1038/s41467-018-04812-6 (PMC6002358; doi:10.1038/s41467-018-04812-6)
Supplement: Supplementary file 1 — Supplementary Information [file 41467_2018_4812_MOESM1_ESM.pdf]

Supplementary Information

Inducing Kondo Screening of Vacancy Magnetic Moments in Graphene with Gating and Local Curvature

Jiang *et al.*

Supplementary Note 1: Electronically decoupled top layer graphene.

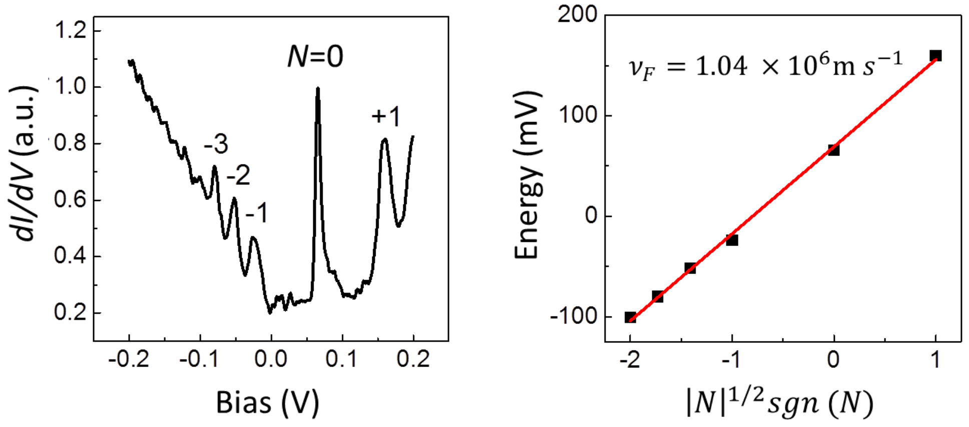

Supplementary Figure 1. (Left) Landau level (LL) spectroscopy of graphene in magnetic field of B = 6T. The numbers label the LL index. (Right) LL peak sequence and its fit to $E_N = E_D \pm \frac{\hbar v_F}{l_B}\sqrt{2|N|}$, $N = 0, \pm 1, ..$ is used to extract the Fermi velocity.

The two graphene layers were stacked with a large twist angle between them to ensure electronic decoupling [1, 2]. This absence of moiré patterns for these samples is consistent with their decoupling. The single layer nature of the top layer is further revealed by the characteristic Landau level (LL) sequence of peaks, $E_N = E_D \pm \frac{\hbar v_F}{l_B}\sqrt{2|N|}$, $N = 0, \pm 1, ..$ which appears in the presence of a magnetic field. Here E_D is the Dirac point (DP) energy measured relative to E_F, v_F is the Fermi velocity and l_B the magnetic length. Supplementary Figure 1 shows the LL spectrum on the graphene surface far from any vacancy from which we extract the Fermi velocity $v_F = 1.04 \times 10^6$ m s^{-1} by fitting to the LL sequence. The specific LL sequence as the fingerprint of massless Dirac fermions is the direct proof of that the two graphene layer are electronically decoupled.

Supplementary Note 2: Criteria to identify the intrinsic single vacancy

As discussed in the main text, several criteria were used to identify the intrinsic single atom vacancies. Firstly, theoretical and experimental work has shown that single atom vacancies display a characteristic triangular structure arising from the electronic reconstruction [3, 4]. This triangular fingerprint is used to distinguish between bare vacancies and vacancies that are passivated by adsorbed adatoms such as hydrogen or nitrogen whose topography lacks this feature [5, 6]. Secondly, the zero mode peak at the Dirac point allows to distinguish between single-atom vacancies and other types of un-passivated defects, such as Stone-wales reconstruction or di-vacancies, which lack both the triangular electronic reconstruction feature and the zero mode in the electronic states [7, 8]. Thirdly, the evolution of the local Dirac point with the distance from the single vacancy center is examined by the dI/dV curves. If a chemical bond forms between the dangling bond and an ad-atom, the electron affinity difference will cause a charge transfer at the vacancy site. This shifts the local Dirac point and gives rise to a clear shift of the Dirac across the vacancy site [9]. Combining all these criteria together ensures the correct identification of single atom vacancies.

Supplementary Note 3: Fano fitting to the Kondo peak

The Kondo temperature is obtained by fitting the *dI/dV* curve to the Fano lineshape $\frac{dI}{dV} \propto \frac{(\varepsilon+q)^2}{1+\varepsilon^2} + A$, where A is the background tunneling signal and q is the Fano asymmetry factor given by $q \propto t_2/t_1$ (t_1 and t_2 are the matrix elements for electron tunneling into the continuum of the bulk states and the discrete Kondo resonance, respectively)[10]. Here $\varepsilon = \frac{eV-\varepsilon_0}{\Gamma/2}$ is the normalized energy (ε_0 is the position of the resonance and Γ is the full width of half maximum (FWHM) of the Kondo peak, which is related to Kondo temperature, T_K, as $k_B T_K = \Gamma/2$). Due to full self-energy of the resonant level interactions and coupling to the conduction electrons, ε_0 may slightly shift away from E_F[11].

Supplementary Note 4: Temperature dependence of the Kondo peak

The temperature dependence of the Kondo peaks is analyzed by raising the temperature from 4.2K to 25K, Supplementary Figure 2.

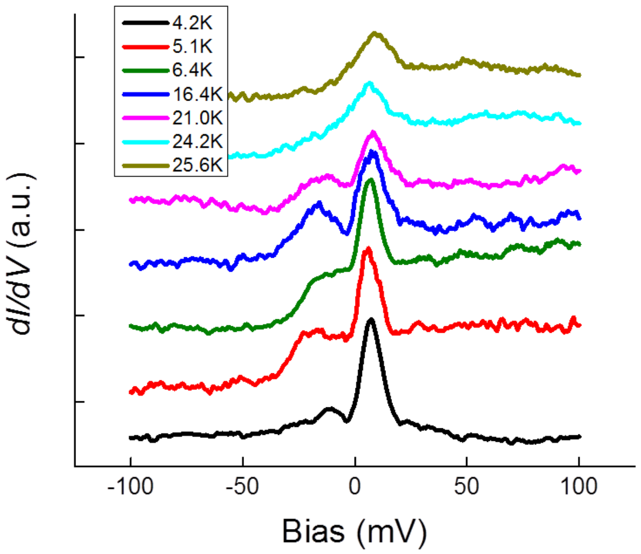

Supplementary Figure 2 Temperature dependence of the Kondo peaks. V_b = -200mV, I = 20pA, V_g = 50V.

Supplementary Note 5: Substrate effect on the Kondo coupling

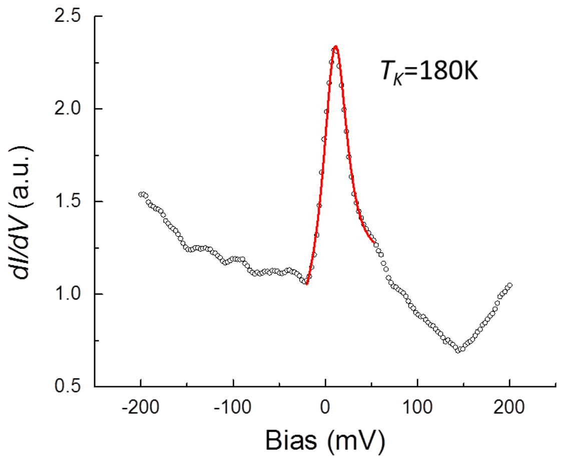

Supplementary Figure 3 *dI/dV* curve together with Fano-fit (red) for a vacancy in G/SiO_2, V_b=-300mV, I = 20pA.

As discussed in the main text and consistent with the theoretical predictions, Kondo screening is observed for the rougher G/SiO_2 and G/G/SiO_2 samples while it is absent on the much smoother G/BN and G/G/BN. Interestingly, most of the earlier work focused on the in-plane Jahn-Teller distortion but not on the out-of plane distortion. This is the first work to show the crucial role of the local curvature. Supplementary Figure 3 shows the Kondo peak for a vacancy in the G/SiO_2 sample where the local curvature is largest. T_K here is much higher than that on G/G/SiO_2 consistent with the rougher surface.

Supplementary Note 6: Pseudogap Anderson impurity model

As discussed in the main text, the interaction of a magnetic moment with the pseudogap conduction band electrons in graphene is captured by the single-channel asymmetric Anderson impurity model (AIM) [12] with the characteristic parameters ε_d, U, and Γ_0 corresponding to the energy of the bare impurity state, the onsite Coulomb repulsion and the coupling strength between respectively.

The Hamiltonian for the AIM can be written as[13]:

$$H = \sum_\sigma \int_{-D}^{D} d\omega \frac{(\omega+\mu)}{D} c_\sigma^\dagger(\omega) c_\sigma(\omega) + \sum_\sigma (\varepsilon_d - \mu) f_\sigma^\dagger f_\sigma + U_{eff}(\mu) f_\uparrow^\dagger f_\uparrow f_\downarrow^\dagger f_\downarrow +$$

$$\sum_\sigma \int_{-D}^{D} d\omega \sqrt{\frac{\Gamma(\omega)}{\pi D}} [c_\sigma^\dagger(\omega) f_\sigma + f_\sigma^\dagger c_\sigma(\omega)], \quad (1)$$

where spin index $\sigma = \uparrow, \downarrow$; D is the conduction bandwidth; $c_\sigma^\dagger(\omega)[c_\sigma(\omega)]$ is the creation (annihilation) operator for an electron in the conduction state with energy ω; μ is the chemical potential; ε_d is the energy of the impurity level; U is the Coulomb interaction between the electrons on the impurity; $\Gamma(\omega)$ is the coupling function; $f_\uparrow^\dagger$ ($f_\downarrow^\dagger$) and $f_\uparrow$ ($f_\downarrow$) are creation and annihilation operators for an electron in the $\uparrow$ ($\downarrow$) impurity state.

Since the zero-mode (ZM) is near the Dirac point [see Fig. 1d and Fig. 1e in the main text] it does not contribute to the Kondo critical behavior in the p-doped regime ($\mu < 0$) as long as the ZM is unoccupied. In the n-doped regime ($\mu > 0$) however when the ZM becomes occupied, the energy of the doubly occupied σ-orbital could rise above the onsite Coulomb interaction U of the σ-orbital due to the additional Coulomb repulsion from the ZM. This increases the

effective Coulomb interaction of the σ-orbital state. Therefore, we take this into account by adopting the following effective Coulomb interaction between the electrons on the impurity:

$$U_{eff}(\mu) = \begin{cases} U & \mu \le 0 \\ U + \min(U_{\pi d}, \alpha\mu) & \mu > 0 \end{cases} \quad (2)$$

where $U_{\pi d}$ is the Coulomb interaction between the ZM and σ-orbital; α is a positive constant. The coupling function can be written as [13]:

$$\Gamma(\omega) = \frac{\Omega_0 V^2 |\omega+\mu|}{2\hbar^2 {v_F}^2}\left[2 - J_0\left(\frac{2}{3}\frac{|\omega+\mu|}{t}\right)\right], \quad (3)$$

where $\Omega_0, V, t,$ and v_F are the unit cell area, the hybridization strength, hopping energy, and Fermi velocity, respectively. We can expand the zeroth Bessel function J_0 at $\omega = 0$, and $\Gamma(\omega)$ can be approximated as:

$$\Gamma(\omega) = \frac{\Omega_0 V^2 |\omega+\mu|}{2\hbar^2 {v_F}^2}\left[1 + \frac{4}{27}\left(\frac{|\omega+\mu|}{t}\right)^2\right]. \quad (4)$$

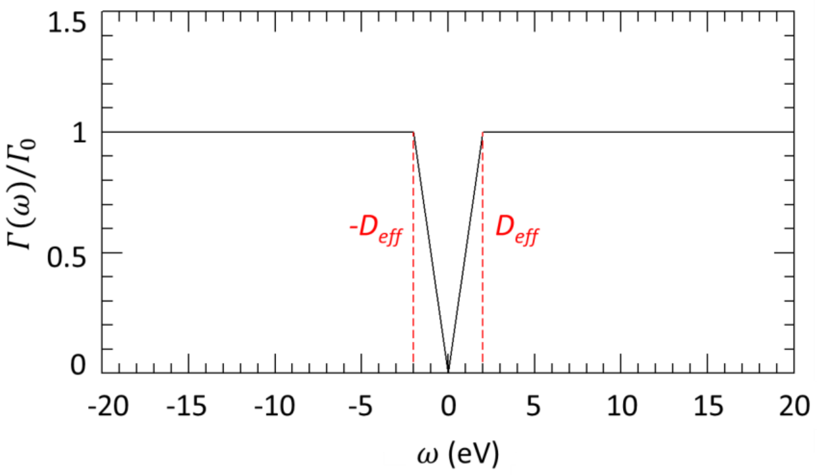

Supplementary Figure 4. The coupling function $\Gamma(\omega)$ adopted in this work. The total bandwidth D = 20 eV and effective bandwidth D_{eff} = 2 eV are taken from the graphene band structure from ab initio density functional calculations.

Earlier, we showed that the second term hardly affects the calculated spectral function[14], and thus we approximate the $\Gamma(\omega)$ as a linear function of ω. In this work, we use the mixed form in which $\Gamma(\omega)$ is proportional to $|\omega + \mu|$ only within the effective bandwidth D_{eff}, and is constant for the rest of the bandwidth (Supplementary Figure 4). Mathematically, $\Gamma(\omega)$ can be written as:

$$\Gamma(\omega) = \begin{cases} \Gamma_0 \frac{|\omega+\mu|}{D_{eff}} & \frac{|\omega+\mu|}{D_{eff}} \leq 1 \\ \Gamma_0 & \frac{|\omega+\mu|}{D_{eff}} > 1, \frac{|\omega|}{D_{eff}} \leq \frac{D}{D_{eff}}, \\ 0 & \frac{|\omega|}{D_{eff}} > \frac{D}{D_{eff}} \end{cases} \quad (5)$$

where $\Gamma_0 = \frac{\Omega_0 V^2 D_{eff}}{2\hbar^2 v_F{}^2}$ represents the coupling strength discussed in the main text.

Supplementary Note 7: Numerical renormalization group calculations

We exploit the powerful numerical renormalization group (NRG) method to solve the Anderson impurity model[15]. In the present calculations, we use the discretization parameter Λ = 1.8 and keep 1200 states per NRG iteration so that the obtained spectral functions converge within 0.1 %. We set the total bandwidth $D = 20$ eV and $D_{eff} = 2$ eV [14] (Supplementary Figure 4). Note that the numerical results do not depend on D and D_{eff} as long as they are larger than 8 eV and 1 eV, respectively. Typically, the onsite Coulomb interaction varies from about 1 to 10 eV. Our experimental results indicate that the singly occupied impurity state (ε_d) is well below the Fermi level and only the doubly occupied impurity state ($U + \varepsilon_d$) is relevant here (i.e., $|U + \varepsilon_d|<|\varepsilon_d|$). Starting with the initial value $U = 2$ eV for the Coulomb interaction strength and $\varepsilon_d = -1.5$ eV for the bare σ-orbital energy [14, 16], we fit several theoretical spectral functions for slightly varied U and ε_d values to a typical experimental *dI/dV* curve, resulting in best fits for $\varepsilon_d = -1.6$ eV and U= 2 eV. With this set of the model parameters, we performed the NRG calculations of the spectral function over a range of Γ_0 values in order to determine the critical value Γ_C which separates the local-moment (LM) and frozen-impurity (FI) regimes at $\mu = 0$. The two regimes are identified by the value of the impurity entropy, S(LM) = ln2 and S(FI)=0 for the LM and FI regimes respectively. We found Γ_C =1.15 eV from the results of these NRG calculations. Finally, by matching the spectral functions to the experimental spectra at different values of the chemical potential (Supplementary Figure 5), we obtain α=3.5 and $U_{\pi d} = 0.2$ eV.

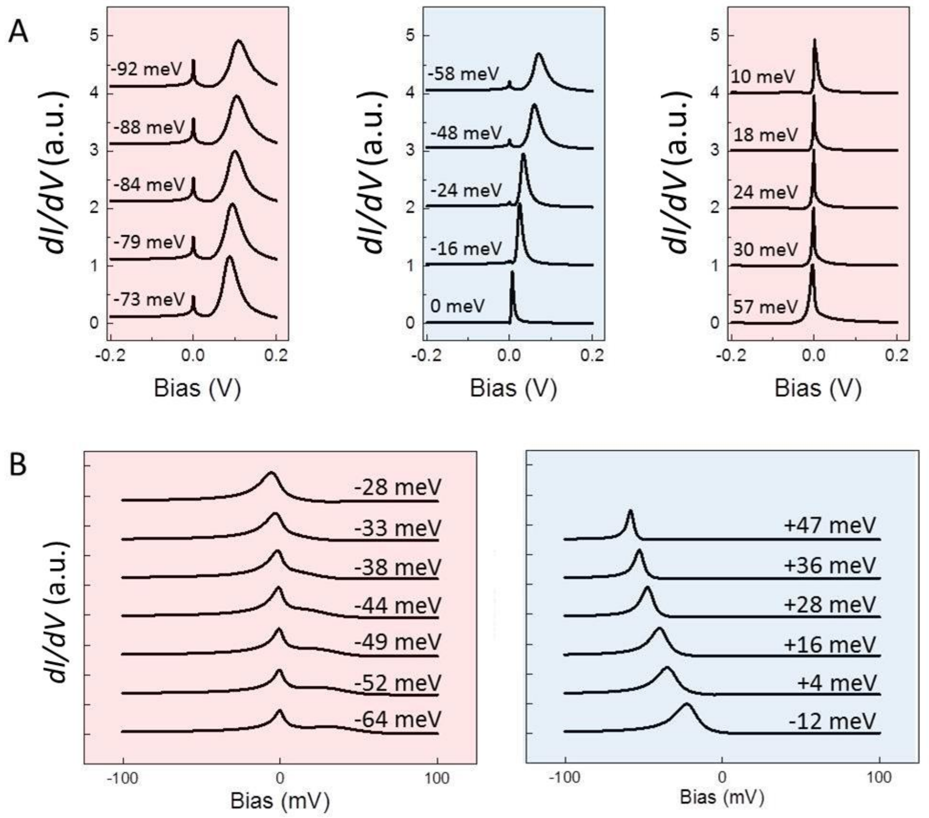

Supplementary Figure 5. Simulated gate-dependent *dI/dV* spectra for (A) $\Gamma_0 = 0.9\Gamma_C$ and (B) $\Gamma_0 = 1.83\Gamma_C$ corresponding to the experimental spectra in Fig. 2 of the main text.

We estimated the Kondo temperatures by fitting the spectral functions calculated at the experimental temperature T = 4.2 K to the Fano lineshape. Fig. 3a shows the Kondo temperature versus the chemical potential for six different vacancies together with the simulated results. For $\Gamma_0 < \Gamma_C$, Kondo screening occurs in both p-doped and n-doped sectors. Supplementary Figure 5 shows the simulated gate-dependent *dI/dV* spectra for (A) $\Gamma_0 = 0.90\Gamma_C$ and (B) $\Gamma_0 = 1.83\Gamma_C$ corresponding to the experimental spectra in Fig. 2 of the main text. The NRG simulations of the vacancy spectra in Fig.2a (main text), correspond to $\Gamma_0 = 0.90\Gamma_C$, which places this vacancy in the subcritical regime. In Supplementary Figure 5B, the NRG simulations of the vacancy spectra in Fig. 2b (main text), correspond to $\Gamma_0 = 1.83\Gamma_C$, which places this vacancy in the supercritical regime. A comparison between the measured (Fig. 3a) and simulated (Fig. 3b) T_K (μ) curves in main text was used to obtain the Γ_0/Γ_C values characterizing each vacancy.

Supplementary Note 8: NRG Phase diagram

The Anderson impurity model has a rich phase diagram where both spin and charge fluctuations play a role. In the case of graphene, where the particle-hole symmetry is broken by the finite value of the next nearest neighbor hopping term or for $U \neq 2|\varepsilon|$, the interaction of the vacancy spin with the linear DOS is captured by the asymmetric Anderson impurity model [17, 18, 19]. The NRG phase diagram for this model is controlled by the valence fluctuation critical point, Γ_c. At charge neutrality, μ=0, Γ_C separates the NRG flow into two sectors: (i) supercritical, $\Gamma_0 > \Gamma_C$, which flows to the ASC fixed point where charge fluctuations give rise to a frozen impurity (FI) correlated ground state. (ii) subcritical, $\Gamma_0 < \Gamma_C$, which flows to the local LM fixed point where the impurity moment is unscreened.

In the supercritical regime, the NRG analysis reveals that the FI is a correlated ground state in which coupling between the impurity and the conduction band electrons is mediated by a cloud of charge fluctuations, resulting in a doubly occupied singlet where spin fluctuations are frozen. In the subcritical regime it was shown [18] that the impurity moment is Kondo screened for any finite value of the chemical potential, $\mu \neq 0$, but at the same time T_K is exponentially suppressed: $\ln T_K \propto -1/|\mu|$. As a result, at sufficiently low doping the value of T_K must fall below any experimentally accessible temperature, so that for all practical purposes its value can be set at 0. As shown in Figure 5 of reference [18], for the experimental parameters relevant to this work (T = 4.2K and $\Gamma < 0.9\, \Gamma_C$), the crossover between screened and unscreened moments as a function of doping is very sharp, which allows us to label the different regimes as distinct phases. The phase diagram presented in Fig. 4A of the manuscript classifies the various regimes at the experimental temperature of 4.2 K using this criterion.

Kondo model. In the limit $|\varepsilon_d|, |U + \varepsilon_d| \gg \Gamma,\ k_B T$ the Anderson impurity model reduces to the well-known spin ½ Kondo model where charge fluctuations are quenched. In this case the single occupancy of the impurity level is overwhelmingly favored over zero or double occupancy, in effect localizing a pure-spin degree of freedom at the impurity site. As a result screening of the impurity moment is mediated by spin fluctuations only. For a linear DOS such as graphene and with a particle-hole asymmetric band, the Kondo model NRG phase diagram is characterized by a critical coupling strength that separates a local moment (LM) phase from

a strongly coupled (ASC) phase where the moment is screened by interactions with the conduction band that are mediated by spin-fluctuations. The dynamics of the Kondo screening cloud is thus distinct from that in the FI where the screening is mediated by charge fluctuations.

Supplementary References

1. Lu C-P, *et al.* Local, global, and nonlinear screening in twisted double-layer graphene. *PNAS* **113**, 113, 6623-6628 (2016).
2. Luican A, *et al.* Single-Layer Behavior and Its Breakdown in Twisted Graphene Layers. *Phys. Rev. Lett.* **106**, 126802 (2011).
3. Amara H, Latil S, Meunier V, Lambin P, Charlier JC. Scanning tunneling microscopy fingerprints of point defects in graphene: A theoretical prediction. *Phys. Rev. B* **76**, 115423 (2007).
4. Banhart F, Kotakoski J, Krasheninnikov AV. Structural Defects in Graphene. *ACS Nano* **5**, 26-41 (2011).
5. Zhao L, *et al.* Visualizing Individual Nitrogen Dopants in Monolayer Graphene. *Science* **333**, 999-1003 (2011).
6. Ziatdinov M, Fujii S, Kusakabe K, Kiguchi M, Mori T, Enoki T. Direct imaging of monovacancy-hydrogen complexes in a single graphitic layer. *Phys. Rev. B* **89**, 155405 (2014).
7. Zaminpayma E, Razavi ME, Nayebi P. Electronic properties of graphene with single vacancy and Stone-Wales defects. *Appl. Surf. Sci.* **414**, 101-106 (2017).
8. Mao J, *et al.* Realization of a tunable artificial atom at a supercritically charged vacancy in graphene. *Nat. Phys.* **12**, 545-549 (2016).
9. Ma C, *et al.* Tuning the Doping Types in Graphene Sheets by N Monoelement. *Nano Lett.* **18,** 386-394 (2018).
10. Fano U. Effects of Configuration Interaction on Intensities and Phase Shifts. *Phys. Rev.* **124**, 1866-1878 (1961).
11. Madhavan V, Chen W, Jamneala T, Crommie MF, Wingreen NS. Tunneling into a Single Magnetic Atom: Spectroscopic Evidence of the Kondo Resonance. *Science* **280**, 567-569 (1998).
12. Gonzalez-Buxton C, Ingersent K. Renormalization-group study of Anderson and Kondo impurities in gapless Fermi systems. *Phys. Rev. B* **57**, 14254-14293 (1998).
13. Kanao T, Matsuura H, Ogata M. Theory of Defect-Induced Kondo Effect in Graphene: Numerical Renormalization Group Study. *J. Phys. Soc. Jpn* **81**, 063709 (2012).
14. Lo PW, Guo GY, Anders FB. Gate-tunable Kondo resistivity and dephasing rate in graphene studied by numerical renormalization group calculations. *Phys. Rev. B* **89**, 195424 (2014).
15. Wilson KG. The renormalization group: Critical phenomena and the Kondo problem. *Rev. Mod. Phys.* **47**, 773-840 (1975).
16. Yazyev OV, Helm L. Defect-induced magnetism in graphene. *Phys. Rev. B* **75**, 125408 (2007).

17. Fritz L, Vojta M. Phase transitions in the pseudogap Anderson and Kondo models: Critical dimensions, renormalization group, and local-moment criticality. *Phys. Rev. B* **70**, 214427 (2004).
18. Vojta M, Fritz L, Bulla R. Gate-controlled Kondo screening in graphene: Quantum criticality and electron-hole asymmetry. *Epl-Europhys. Lett.* **90**, 27006 (2010).
19. Fritz L, Vojta M. The physics of Kondo impurities in graphene. *Rep. Prog. Phys.* **76**, 032501 (2013).
